# BatchMap: A parallel implementation of the OneMap R package for fast computation of F_1_ linkage maps in outcrossing species

**DOI:** 10.1371/journal.pone.0189256

**Published:** 2017-12-20

**Authors:** Bastian Schiffthaler, Carolina Bernhardsson, Pär K. Ingvarsson, Nathaniel R. Street

**Affiliations:** 1 Umeå Plant Science Centre (UPSC), Department of Plant Physiology, Umeå University, Umeå, Sweden; 2 Department of Ecology and Environmental Science, Umeå University, Umeå, Sweden; 3 Department of Plant Biology, Uppsala BioCenter, Swedish University of Agricultural Sciences, Uppsala, Sweden; Universidad Miguel Hernández de Elche, SPAIN

## Abstract

With the rapid advancement of high throughput sequencing, large numbers of genetic markers can be readily and cheaply acquired, but most current software packages for genetic map construction cannot handle such dense input. Modern computer architectures and server farms represent untapped resources that can be used to enable higher marker densities to be processed in tractable time. Here we present a pipeline using a modified version of OneMap that parallelizes over bottleneck functions and achieves substantial speedups for producing a high density linkage map (N = 20,000). Using simulated data we show that the outcome is as accurate as the traditional pipeline. We further demonstrate that there is a direct relationship between the number of markers used and the level of deviation between true and estimated order, which in turn impacts the final size of a genetic map.

## Introduction

Genetic linkage maps are constructed to determine the relative order and distance between loci on a chromosome. These maps can, among other things, be used for association genetics and marker assisted breeding by linking phenotypic traits to regions and genes in the map (QTL mapping), to improve fragmented genome assemblies by ordering and orienting scaffolds along chromosomes [1, 2], or to analyze genome synteny between related species [2]. However, constructing a genetic map is often a great challenge due to the computational effort involved and, until recently, the developmental cost and time investment required to identify reliable markers. Next generation sequencing approaches have removed the barriers to identifying and subsequently assaying large number of markers with genome-wide coverage, enabling most, if not all, genes to be placed on a genetic map. Creating reliable, high density linkage maps is therefore rapidly becoming an even greater computational challenge as marker density has rapidly increased. The computational challenge arises from bottlenecks involved in determining the order of markers (a traveling salesman problem) and the optimization of recombination frequencies and phasing (using the EM-algorithm, [3]). An exhaustive search for the true order requires n!/2 comparisons (n = number of markers on the LG) and with anything more than 10 markers this calculation rapidly becomes computationally prohibitive.

In order to deal with these computational problems, several heuristic approaches for marker ordering have been developed. One of the most broadly employed programs (that implements a regression- and a maximum likelihood mapping algorithm) for constructing linkage maps is JoinMap [4], a commercial, proprietary license based, Microsoft Windows-only software. However, during the last decade a number of open source software implementations, including OneMap [5], have been developed (e.g. [6], [7], [8], [9], 10], see also [1] for a summary table). All of these have implemented both different ordering algorithms and mapping functions. All softwares have their unique benefits and drawbacks, but unfortunately they are often computationally slow as they do not utilize parallel processing, are often written in such a way that it is hard to follow the process and confirm the results, are available as online softwares only, or are designed to work with only a limited range of the most common pedigree designs (e.g. RILs, F_2_s). For researchers working with organisms with long generation time (e.g. forest trees or large mammals with generation times of 15 to 20 years), it may be demanding to create an F_1_ and infeasible to create an F_2_ or backcross. When working with F_1_ crosses, where no linkage information between markers is availible from the parents and several different phases per marker pair need to be considered, the double pseudo-testcross approach [11] has proven effective: separate maps are created for the two parents, with markers that are heterozygous in both parents providing links between the two maps. As two maps are created, this approach requires twice the number of LGs to be analyzed compared to RILs, backcrosses or F_2_s. However, the availability of large server clusters, either within research groups, through national- or international infrastructures or commercial cloud computing services, could be utilized to decrease real-world computational time by implementing parallelization of several of the time consuming steps in linkage map construction.

OneMap is an open source package for the R programming language [12] that implements all ordering algorithms and mapping functions available in the proprietary JoinMap program. It is well documented, with extensive guidelines for users on map creation and is also under active development via GitHub (https://github.com/augusto-garcia/onemap). However, it has not currently implemented an option for parallelization of the analyses and is therefore prohibitively slow when working with large numbers of markers. Here, we describe our extended OneMap implementation, BatchMap, which comprises faster versions of three ordering and mapping function algorithms:

1. record.parallel(): RECORD [13] is a popular heuristic approach to approximate the true order of markers. Both the RECORD algorithm and Wu et al.’s EM optimization for phasing and recombination fraction (RF) computation have previously been shown to perform exceptionally well in full-sib outcrossing mapping populations with relatively noisy datasets containing up to 20% missing genotypes per marker [14].
2. map.overlapping.batches(): A mapping algorithm that splits a pre-ordered LG into batches containing a predefined number of overlapping markers between neighboring batches. Here, batches are analyzed in sequential order, with overlapping markers fixed in phase from the previous batch.
3. ripple.ord(): An algorithm inspired by the OneMap ripple.seq() function, which leverages the high speed of the batch mapping approach in addition to multiple CPUs to consider alternative marker orders in parallel to improve the order and reduce errors (hereinafter called ‘ripple’).

By using these approaches we can create a high density linkage map within tractable time scales (days) as opposed to months or years, depending on the computational power available for the project and the number of markers used in the map. Our OneMap fork ‘BatchMap’, the example data and user manual is freely available for download from: https://github.com/bschiffthaler/BatchMap.

## Methods

### Simulated data

In order to evaluate the performance of the BatchMap algorithms, we created four simulated F_1_-crosses using QMSim (QTL and Marker Simulator) version 1.10 [15]. Each cross was replicated once with an historical population size of 1000 individuals per generation for 1000 generations before the cross was made. Each of the genomes was set to have five chromosomes with a chromosome length (chrlen) of 200 cM and 1500, 2000, 3000 and 4000 markers per chromosome (nmloci) for the four crosses, respectively. This resulted in four datasets containing 7500, 10000, 15000 and 20000 markers, all with a total genome size of 1000 cM (200cM per linkage group for each of the datasets). QMSim uses the chrlen in Morgans as the mean for the Poisson distribution when calculating cross-over during meiosis, resulting in the final maps calculated from the simulated crosses being half the size of the pre-set value (in our case ^~^100 cM per chromosome). All data sets were set to include only biallelic markers (nma) positioned randomly in the genome (mpos) and with marker allele frequencies (maf) of 0.5, but with a rate of missing data per genotype (rmmg) of 0.2. Due to the simulated nature of the data, it was not filtered for segregation distortion. We advise using a *χ*^2^ test to filter distorted markers in real datasets. A random mating design (md) and a litter size (ls) of 800 with a 50/50 ratio of female and male progeny was then set to create each of the F_1_ crosses. The simulated data sets were thereafter filtered so that only informative markers were kept (markers for which none of the parents had missing data and where at least one of the parents was heterozygous), and transformed into OneMap input format for either of the cross types “B3.7” (*ab x ab*), “D1.10” (*ab x aa*), “D2.15” (*aa x ab*) or “-” according to the encoding scheme in [3]. This resulted in final data sets containing 2368, 3042, 4714 and 6182 markers, respectively (Table 1 and Supplementary Files inside S1 Simulated Data).

**Table 1:** Summary of the simulated data sets. Data set: The name of the data set; Markers/chr: Number of markers simulated on each chromosome; Total markers: The total number of markers; Genetic map: Number of markers in the genetic map after filtering for informative markers (and the corresponding percentage of all simulated markers); Markers/LG: The average number of markers on each LG (and the marker density range).

Data set	Markers / chr	Total markers	Genetic map	Markers / LG
Sim7.5k	1 500	7 500	2 368 (31.6%)	471 (451-489)
Sim10k	2 000	10 000	3 042 (30.4%)	608 (580-627)
Sim15k	3 000	15 000	4 714 (31.4%)	943 (916-967)
Sim20k	4 000	20 000	6 182 (30.4%)	1 236 (1 196-1 280)

### BatchMap pipeline

#### Data input and preparation

**Fig 1:**
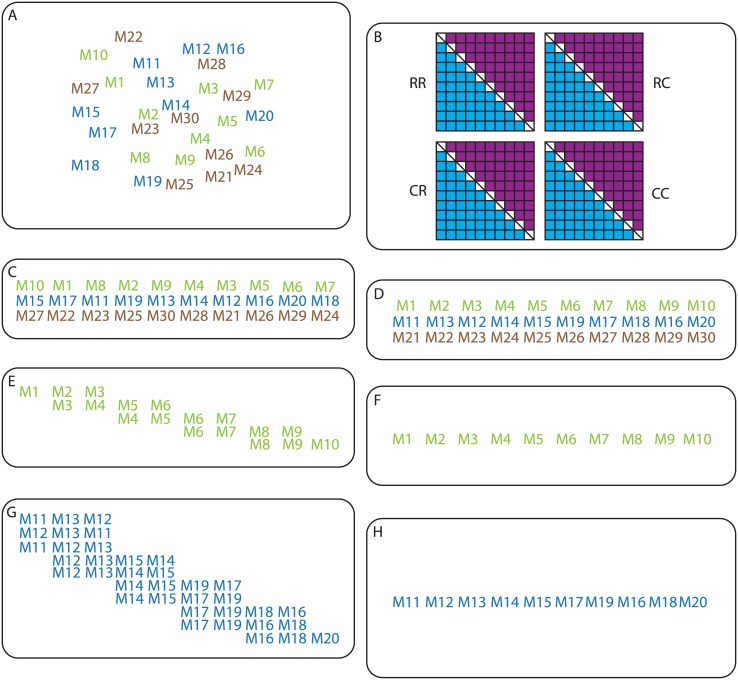
An overview of the BatchMap pipeline. **A**) Input data file is read into BatchMap using the function read.outcross2(). **B**) A two-point table of recombination fractions is created and likelihoods calculated per phase using the function rf.2pts(). **C**) Markers are assigned to linkage groups (LGs) using the function group(), sequence objects created using make.seq() and LGs are split into parental maps using marker segregation type. **D**) Marker order is determined using RECORD, with the number of iterations and threads set using the function record.parallel(). **E**-**F**) The genetic map is calculated in centiMorgans by first using the function pick.batch.sizes() to split the LG into even groups and then using function map.overlapping.batches() with the predefined batch size. All pairwise marker phases are set within each batch and overlapping markers are fixed in phase from the previous batch. When all marker pairs are fixed in phase the original OneMap function map() is called to calculate the distance between markers in cM using either the Kosambi or Haldane mapping function. **G**-**H**) An optional ripple function, ripple.ord(), can be set in map.overlapping.batches() to consider all single pairwise swaps between markers in a sliding window of predefined size along the LG. When the best order (based on likelihoods) within each window is set, map calculation will be carried out as in E-F).

Fig 1 shows a flowchart of the important steps described in this section. An example R script is available as BatchMap.R inside S1 R Scripts. The simulated data (see Supplementary Files inside S1 Simulated Data) was read into BatchMap using the function read.outcross2(), which was added as the original function in OneMap was not suited for large datasets. The next steps follow the default OneMap workflow in creating the two-point tables of recombination fractions and likelihoods (rf.2pts()) and assigning the markers to linkage groups (group()) using LOD = 8 and max.rf = 0.35. Sequence objects were created for each linkage group (make.seq()), which were then split into parents by the marker segregation type. The segregation type D2.15 and B3.7 were assigned to parent one, whereas segregation type D1.10 and B3.7 were assigned to parent two.

#### Marker ordering

RECORD (recombination count and ordering) works by adding one random marker at a time and orders markers by minimizing the number of recombination events in the map. This is replicated *x* number of times and the order with the lowest COUNT criterion, which minimizes the number of recombination events needed to explain the marker order, (see eq. 1 in Van Os et al. 2005) is accepted as the true order of markers. We implemented a parallelized version of the RECORD algorithm that utilizes multithreaded computing to calculate the marker order using RECORD *N* times in parallel (record.parallel()). As each iteration is an independent calculation, this is trivial to parallelize. We opted to perform ten iterations, as increasing the number beyond that did not appreciably improve marker order (see Fig B in S1 Appendix).

#### Batch map

OneMap utilizes an expectation maximization (EM) algorithm to estimate phases and true recombination frequencies in constructing the linkage map (map()). It starts by calculating the likelihood for each possible phase of the first marker pair in a sequence of markers, then iteratively adds the next marker pair and calculates the likelihood of their phase and recombination fraction. For each added pair, all previous data is considered in the model. While this yields the best possible model, long sequences can become virtually impossible to compute. We therefore implemented a new function to calculate the linkage map in overlapping batches, such that enough data for sensible estimation of phases and recombination fractions via the EM model is available in each batch, while also keeping the data from previously evaluated pairs limited. Important parameters for the pipeline are number of markers in a batch (“batch size”) and the number of markers overlapping between adjacent batches (“overlap size”). The EM algorithm will run *N* − 1 times for *N* markers in a batch, and it will consider the result of previous pairs in each iteration. This information is necessary for accurate calculation of phase likelihoods, but the return saturates quickly, compliating the model while offering no additional accuracy. BatchMap divides the markers into batches and only carries a set amount of phase information over to subsequent batches, circumventing this issue. For a given target batch and overlap size, the function pick.batch.sizes() selects a batch size that splits the data into even groups. The function map.overlapping.batches() then considers the batches, but estimates likelihoods for all phases in parallel. Once finished, it will call the original OneMap function map() with predefined phases and order. This approach enables us to reduce the time required to calculate an un-phased map. By dividing the calculation into several sequential sub-problems we achieve comparable accuracy in substantially less time while also providing nearly linear scaling (see section 1 of S1 Appendix), rendering the approach feasible for extremely high-density maps. The simulated data was run with a batch size of 50 markers and an overlap of 30 markers using four CPU threads to consider phases in parallel (see BatchMap.R inside S1 R Scripts).

#### Batch map with adaptive re-ordering via ripple

All ordering algorithms for genetic linkage maps are heuristic approximations since the mathematical problem is NP-complete. This leads to errors in the marker orders generated from these. In order to improve the marker order as much as possible, the function map.overlapping.batches() can be supplied with an ordering algorithm, which makes it possible to consider alternative orders in the calculation of the map. We implemented the function ripple.ord() to provide an algorithm that creates windows of a specified window-size along the markers of a batch and swaps markers according to a set of rules. By default (ruleset ‘one’), all single pairwise swaps between markers are considered. For a given window, all permutations are also calculated in parallel. For any batch besides the first, the part that overlaps the previous batch is exempt from this consideration as not enough information is available to confidently select good alternative orders. The simulated data was run with a window size of five markers, using ruleset ‘one’, and utilizing eight threads to calculate marker permutations in parallel (see BatchMap.R inside S1 R Scripts).

### Currently available OneMap versions

As a comparison we used the current stable version (2.0-4) of OneMap available on CRAN (https://cran.r-project.org/web/packages/onemap/index.html) and the development version (SHA: 53352d13) available on GitHub (https://github.com/augusto-garcia/onemap) on the same data with the same settings for all functions that are available in these versions (see CRAN.R and GitHub.R inside S1 R Scripts).

### Performance evaluation

Both time and accuracy were considered as important factors in evaluating the performance of each run of OneMap. To obtain a reliable estimate for high density maps, we used the first three chromosomes (6 LGs), without binning, from the sim20k dataset for the analyses. Each of these LGs contained between 725 and 794 markers.

The biggest bottleneck for the analysis is the time required to calculate the linkage map phases and recombination fractions, which was therefore selected as the representative time for each run. Further, the number of markers in a sequence impacts the total time required for linkage map construction. The time was therefore compared as the mean time of five replicate runs divided by the number of markers. As map accuracy is dependent on a number of factors, such as the two-point table, linkage group members and the order supplied by RECORD, these were controlled by generating linkage groups and an initial order for the dataset and loading those, such that all runs had an equal entry point into map() or map.overlapping.batches(). The map size in centiMorgans and the overall likelihood reported by OneMap were both considered in the accuracy analysis.

To evaluate whether the ripple algorithm improves order we tested runs for all versions from raw data as well as from a controlled start point (RECORD order). Three measures of order were computed: First, an overall error rate as the rate of markers that were not at their true position. Second, a weighted error rate as the sum of absolute distances of markers from their true position normalized by the length of the sequence (See section 2 of S1 Appendix). Thirdly, Kendall’s tau of the ordered sequence versus the true order.

### Order accuracy vs marker density

In order to evaluate how the number of markers on an LG affected the order accuracy from RECORD and, in turn, to estimate the window size needed for ripple to correct the errors, all five pseudo-testcross chromosomes (10 LGs) from each of the four simulated data sets, after binning, were analyzed. Each map was ordered using 33 iterations of RECORD using record.parallel() using 11 CPU cores and calculated with map.overlapping.batches(), where batch size was set from the pick.overlapping.batches() function with a batch size of 40-60 and 30 overlapping markers. Ordering was carried out before it was determined that running RECORD > 10 times yields no significant benefit, but it was deemed unnecessary to re-do the order as no significant differences are to be expected. Order accuracies were obtained as previously stated by calculating distance from true position for each marker and the total number and distance of mispositioned markers in the maps. Correlations between estimated order and true order were calculated with Kendall’s tau. Map size inflations were also analyzed between the true order maps and the estimated maps from BatchMap record.parallel() and map.overlapping.batches() and the correlation between size inflation and marker density was calculated with Pearson’s product-moment correlation.

## Results

### BatchMap is significantly faster than OneMap

**Fig 2:**
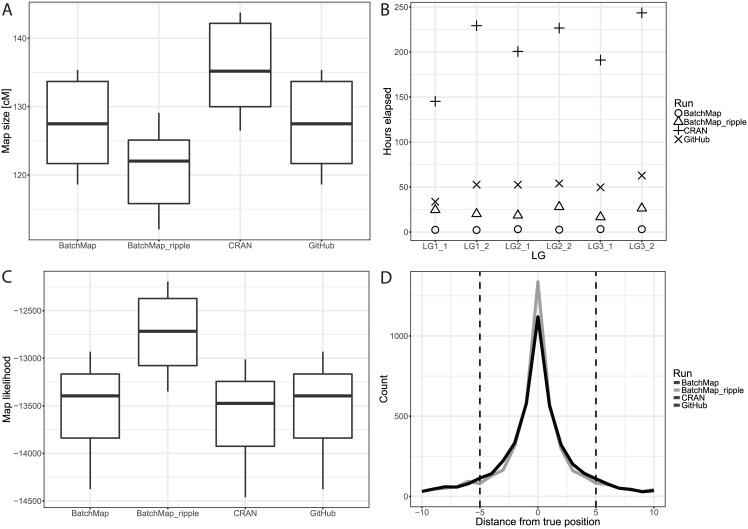
Summary statistics of all evaluation runs for OneMap and BatchMap. In order to control for random effects during ordering and grouping, those factors were estimated once using RECORD and supplied to all runs. **A**) Boxplots (N = 6) of linkage map sizes in centiMorgans for all evaluations. **B**) Hours elapsed per calculation for each pseudo-testcross of three linkage groups between 724 and 794 markers in density. **C**) Boxplots (N = 6) of linkage map likelihoods for all evaluations. **D**) Frequency polygons for all evaluations summarizing the distance of each marker to its true position. All versions except BatchMap_ripple are identical and hence overlap. BatchMap_ripple was run with a window size of five, which is indicated by the vertical dashed bars.

By using a simulated dataset containing over 1,000 markers per chromosome and over 700 markers for each pseudo-testcross we show that the BatchMap is as accurate as the original version of OneMap but substantially faster for high-density maps. We further show that adaptive re-ordering of markers within batches improves map accuracy while still being faster than the original implementations. Calculating full linkage maps for three linkage groups in each of two parents (six maps) with all versions of OneMap yielded a mean processing time per marker of 964.55s for the stable version of OneMap available on the Comprehensive R Archive Network (CRAN), 237.97s for the development version on GitHub (commit 53352d1, June 23rd 2016), 13.19s for our parallel implementation at 16 parallel CPUs and 105.12s for the parallel ripple method when considering all pairwise marker swaps in a sliding window of size 5 (see Fig 2b). Mean total runtimes were 206.06h, 50.92h, 2.82h and 22.42h respectively. Testing was performed on an HP ProLiant DL585 G7 server with four AMD Opteron 6386 SE CPUs and 512 GiB RAM running Ubuntu 16.04 server edition.

### The new implementations perform as well or better than the original

Due to the controlled entry point (marker order derived from 10 iterations of RECORD) for accurate comparison of map() and map.overlapping.batches() between OneMap and BatchMap versions, CRAN, GitHub and BatchMap all showed equal error rates, weighted error rates and Kendall’s tau (Figs C, D and E in S1 Appendix). BatchMap_ripple, with a five marker window size, reduced the mean error rate from 0.76 to 0.71, the weighted mean error rate from 0.016 to 0.015, the median distance to true marker position from 2 to 1 (Fig 2D). Both GitHub and BatchMap resulted in equal final map sizes (118.62cM to 135.37cM) and map likelihoods (-14375.56 to -12931.60) for all six LGs, while performance of the CRAN version was worse (map size 126.48cM to 143.76cM and map likelihood -14461.11 to -13012.67). BatchMap_ripple outperformed the other methods by reducing the size inflation (map size 112.06cM to 129.10cM) and having higher likelihoods (-13349.51 to -12194.68) (Fig 2A and 2C).

### Marker density of a map defines the window size needed for ripple

**Fig 3:**
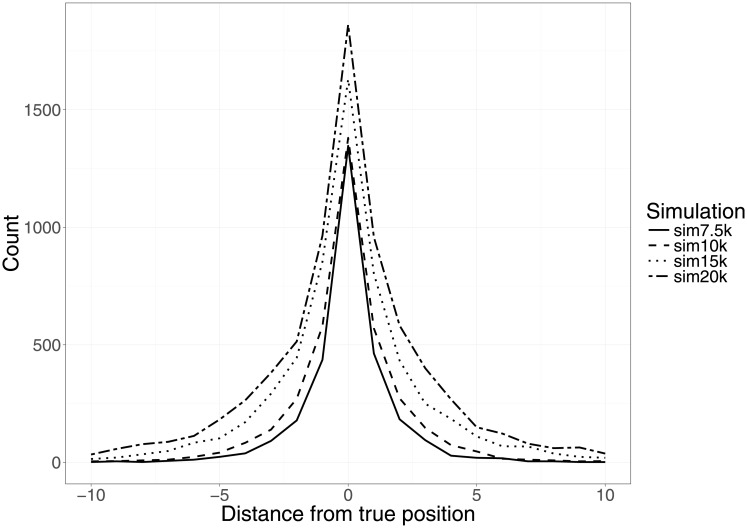
Accuracy of estimated marker order (using 33 iterations of RECORD) for the four data sets. Distribution of distance from true position summed over 10 LGs each for sim7.5k, sim10k, sim 15k and sim20k.

To estimate the effect of marker density on order accuracy, all five chromosomes (10 LG) from each of the four simulated datasets (40 LGs in total) were analyzed with record.parallel(times = 33, …). While the LGs showed a threefold difference in marker density between low and high density LGs (266—777 markers/LG), the number of incorrectly positioned markers increased by 4.5 times (134—608) and the error rate increased from 0.48 to 0.78 (Table A in S1 Appendix). The total distance from true positions showed an almost ninefold increase between low- and high density maps (260—2310). This resulted in an average distance from the true positions of 1-3 markers when calculated over all markers in the LG, and 1.5-4 markers when calculated over only the mis-positioned markers (Table A in S1 Appendix, Fig 3). All LGs showed a correlation of 0.986-0.993 between true and estimated marker order, which was not dependent on marker density (t = -0.431, df = 38, p-value = 0.669). Size inflation (cM) of the estimated maps was highly correlated with marker density (*ρ* = 0.717, t = 6.333, p-value = 1.992e-07).

## Discussion

BatchMap performs the ordering and map calculations at a substantially increased speed compared to both the CRAN (73 times faster) and GitHub (18 times faster) versions of OneMap without lowering the accuracy of the final map. Even when utilizing the ripple function, BatchMap is nine and two times faster than CRAN and GitHub, respectively, but increases the accuracy of marker order within the predefined window size. In order to correct as many marker ordering errors as possible, the window size needs to be larger than the maximum distance to true position that exists in the LG. This distance varied considerably between different RECORD runs regardless of marker density, but no marker in the 40 analyzed LGs had a maximum distance greater than 25 positions away from its true location (Table A in S1 Appendix). However, with increasing map density, a greater portion of the mispositioned markers will be further away from their true location (Fig 3). This means that as map density increases, correspondingly larger window sizes are needed in order for ripple to correct the same proportion of the markers.

The pipeline is mainly controlled by the “batch size” and “overlap size” parameters, which respectively set the number of markers in a batch and the number of shared markers between adjacent batches. If the batch size were set too high, there would be less gain in execution time. If the overlap size would be too small, phases would be incorrectly estimated and large gaps would appear in the map, inflating its size. In practise, these values will depend on many factors such as population size, marker quality and species. It is therefore recommended to try several configurations on a subset of the data and select the best performing one (e.g. best likelihood, smallest size).

Due to the way marker order and phases are locked in the overlapping part of neighboring batches when running map.overlapping.batches(), ripple.ord() will not be able to swap marker order in the area joining the previous batch. To overcome this issue, two or more runs of BatchMap could be performed with different batch sizes in each run. The batch size in the second run should be a ripple window size shorter than the first run in order to release the last fixed markers for swapping. This approach could also be utilized for iteratively decreasing the maximum distance of markers to their true location and thereby fitting them into the ripple window and fixing the errors. The datasets analyzed in this study were simulated with 20% missing data to mimic natural crosses that will always, to some extent, suffer from missing data and genotyping errors. It is important to keep in mind that the cleaner a dataset is, the more accurate the resulting map will be (garbage in, garbage out). Both missing data and genotype errors may falsely link markers together due to missed or extra erroneously inferred recombination events [16]. Fundamentally, the resolution of the genetic map is defined by the number of individuals that comprise the mapping population. Using more markers than the resolution of the mapping population will yield groups of markers with identical information content. A binning step—i.e. using only one representative marker from a group—was omitted in the performance evaluation in order to maximize the number of markers which were input into the algorithms, but should almost always be applied in an experimental setting. This is especially important in smaller mapping populations, where the number of individuals used might not offer a high enough resolution for the marker density that was obtained for the linkage map. OneMap offers the functions find.bins() and create.data.bins() for this purpose.

Marker quality is a crucial factor, which determines map accuracy, but high throughput techniques are often noisy. Low quality markers will increase the run-time for little to no accuracy gain at best, and a higher error rate at worst. In addition to filtering the data for markers with much missing data in the offspring or any missing data in the parents, it is advisable to filter for segregation distortion.

The time required to order markers on an LG with RECORD is determined by the number of runs defined and the number of markers on the LG. Our parallel implementation splits runs into parallel sessions so that they can be performed simultaneously, with runtime depending on the number of cores assigned. Ten runs split over five cores will provide a theoretical five fold speedup in comparison to the CRAN and GitHub versions, while 10 cores will give a theoretical 10 fold speed up. The real advantage of the new parallel implementation is, however, in the map batches approach. While the CRAN and GitHub versions of OneMap show a triangular increase in the time required to calculate the map (see section 1 of S1 Appendix), BatchMap increases quasi linearly with marker density (Fig A in S1 Appendix). If one were to create a full linkage map of a dataset with similar density than the simulated 20k data (^~^1300 markers per chromosome, ^~^750 markers per pseudo-testcross) for an organism with 10 chromosomes, the CRAN version would take 171.7 days, the GitHub version 42.4 days and BatchMap 2.4 days.

## Supporting information

S1 Appendix Additional accuracy and time statistics. (PDF)

S1 Simulated Data Contains simulated data for the manuscript containing. sim7.5k: Simulated markers for this study (7.5k data set)sim10k: Simulated markers for this study (10k data set)sim15k: Simulated markers for this study (15k data set)sim20k: Simulated markers for this study (20k data set).
(ZIP)

S1 R Scripts Contains R scripts used in the manuscript. BatchMap.R: R script for the calculation of linkage maps using BatchMapCRAN.R: R script for the calculation of linkage maps using OneMap (CRAN)GitHub.R: R script for the calculation of linkage maps using OneMap (GitHub).
(ZIP)
