# Supplementary material for: BatchMap: A parallel implementation of the OneMap R package for fast computation of F1 linkage maps in outcrossing species
Source: PLoS One. 2017 Dec 20;12(12):e0189256. doi: 10.1371/journal.pone.0189256 (PMC5738033; doi:10.1371/journal.pone.0189256)
Supplement: S1 Appendix — (PDF) [file pone.0189256.s001.pdf]

Supplementary information: BatchMap: A parallel implementation of the OneMap R package for fast computation of linkage maps in outcrossing species

1 The scaling of BatchMap is nearly linear

In order to evaluate how well `map.overlapping.batches()` scales with the number of markers, five subsets of LG1 (sim20k) were generated in sizes increasing by a factor of two: 50, 100, 200, 400, 800. The maps were then generated using a batch size of 40 and an overlap of 25 (4 parallel phase cores). The total time it took from calling `map.overlapping.batches()` was aggregated and the scaling ratio determined. With each duplication of marker number, the time increased by a factor of 2.38, 2.13, 1.82 and 2.29 respectively. While this is not a comprehensive algorithm analysis, this indicates near linear scaling as N increases. Specifically, the number of times the EM algorithm has to be called in OneMap scales triangular with marker number N as the triangular number of N (equation 1, Supplementary Figure A), while the scaling of BatchMap depends on the number of batches B, the overlap of markers between batches o and the number of markers in a given batch b (equation 2, Supplementary Figure A).

$$\frac{N \cdot (N+1)}{2} \tag{1}$$

$$\frac{b_1 \cdot (b_1+1)}{2} + \sum_{i=2}^{B} \frac{(b_i - o) \cdot (b_i - o + 1)}{2} + o \tag{2}$$

2 Calculation of error rates

The error rate is calculated as the sum of misplaced markers e over the length N of the sequence (equation 3). The weighted error rate is calculated as the absolute distance b of a marker to its true position over the maximally possible disorder given by the triangular number for the length N of the sequence (equation 4).

$$\frac{\sum e_i}{N} \tag{3}$$

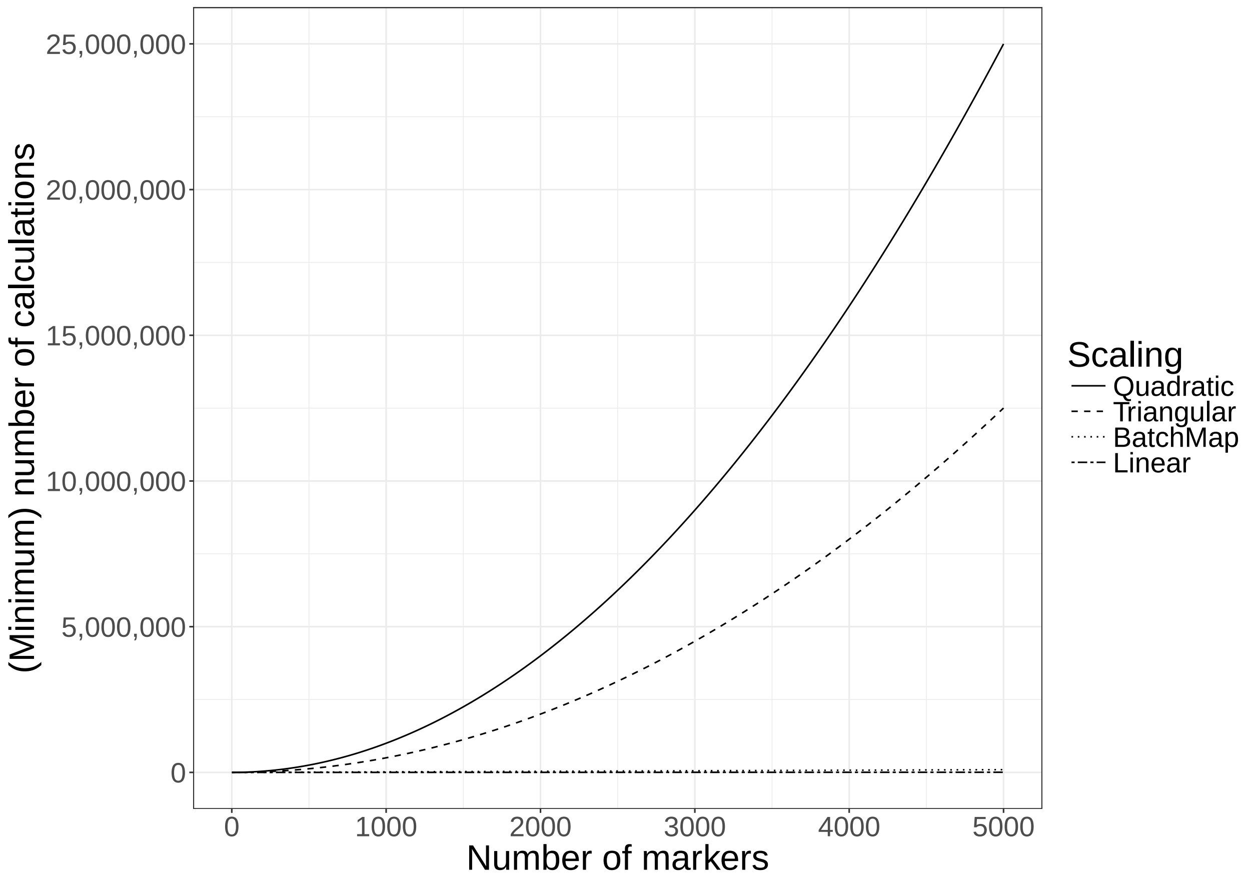

Figure A: Naïve calculation of scaling rates for a quadratic algorithm, a triangular algorithm (such as the phase estimation in OneMap), a linear algorithm and the BatchMap algorithm.

$$\frac{1}{\sum |d_i|} \cdot \frac{N \cdot (N+1)}{2} \tag{4}$$

3 The number of RECORD iterations has little return after the first few

RECORD was executed nine times for two pseudo-testcrosses of five linkage groups (N=10) from the sim20k dataset, each time setting the number of iterations to the next power of two ($[1, 2, 4, 8 \dots 256]$). Each time, the following statistics were calculated: Kendall's tau (compared to true order), error rate (equation 3), weighted error rate (equation 4), mean distance of each marker to its true position, median distance of each marker to its true position. We found that even at two iterations, the results rarely improve much (Supplementary Figure B) and recommend the use of ten iterations as a safe choice.

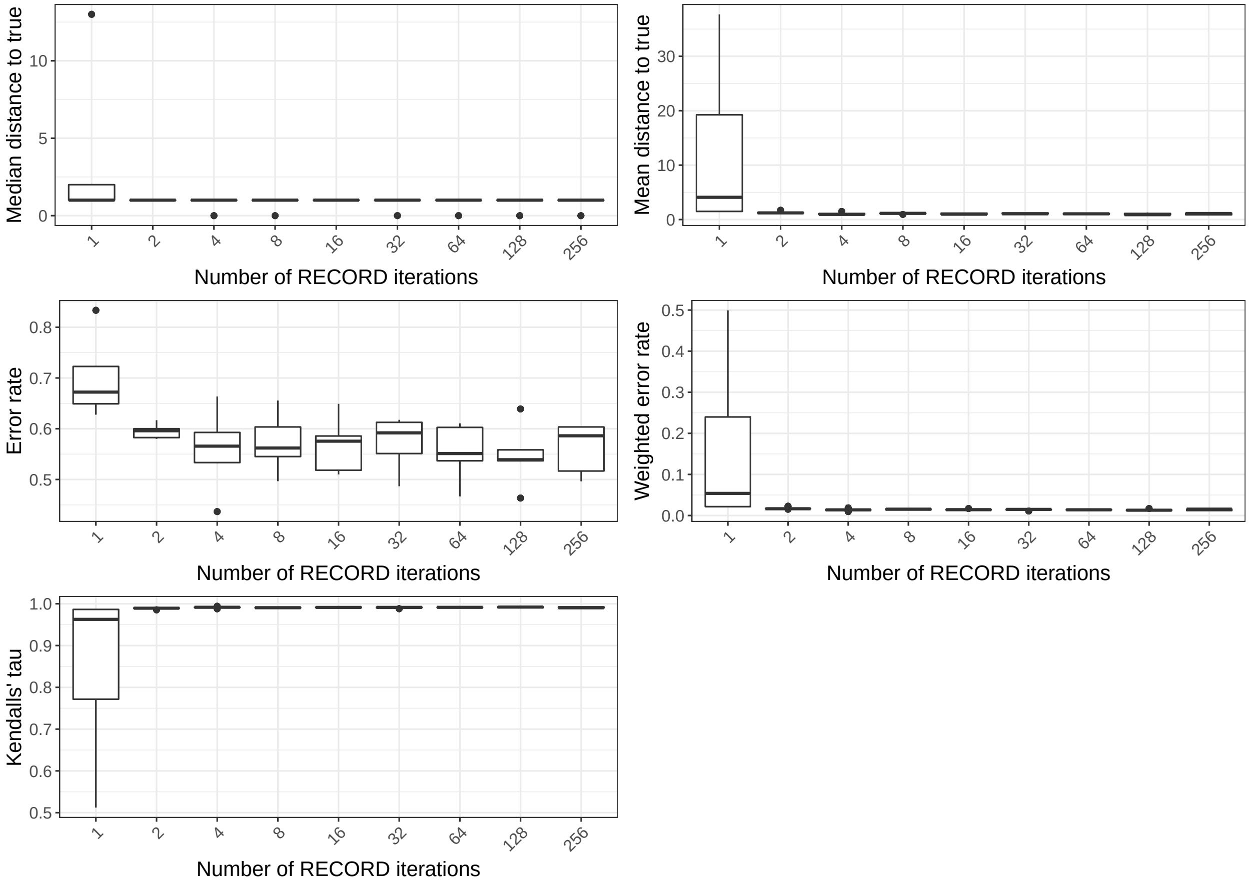

Figure B: Order accuracy statistics for varying values of RECORD iterations (five LGs, two pseudo-testcrosses each, $N = 10$).

4 Additional accuracy statistics for evaluation runs

Additional basic statistics besides likelihood, size and order were collected for all evaluation runs. These are: Kendall's tau (compared to true order), error rate (equation 3) and weighted error rate (equation 4).

Table A: Ordering accuracy for 40 LGs using 33 iterations of RECORD. LG: Name of the pseudo testcross LG; Markers: Number of markers on the LG; Bins: Number of unique bins on the LG; Corr: Correlation between true and estimated marker order calculated with Kendall's tau: Wrongly positioned markers: Number of markers that have been wrongly positioned; Total distance: The distance away from true position summed over all markers; Max distance: The maximum distance away from true position encountered on the LG; Average distance: Total distance over the number of bins on the LG; Average distance (weighted): Total distance over the number of wrongly positioned markers; Size inflation (cM): The difference in size in centiMorgan between estimated and true order LGs. Two horizontal lines indicate the border between datasets sim7.5k, sim10k, sim15k and sim 20k, respectively.

LG	Markers	Bins	Corr	Wrongly positioned markers	Total distance	Max distance	Average distance	Average distance (weighted)	Size inflation (cM)
LG4.1	274	266	0.991	134	260	14	0.977	1.94	8.61
LG2.1	285	279	0.991	161	274	6	0.982	1.702	8.52
LG4.2	303	295	0.993	162	252	5	0.854	1.556	8.82
LG2.2	302	296	0.991	159	298	8	1.007	1.874	10.31
LG5.2	298	296	0.991	158	308	7	1.041	1.949	9.31
LG3.2	304	298	0.992	164	290	18	0.973	1.768	11.61
LG5.1	302	300	0.99	178	374	9	1.247	2.101	10.72
LG3.1	300	300	0.992	145	284	9	0.947	1.959	10.08
LG1.2	318	312	0.991	177	332	8	1.064	1.876	13.09
LG1.1	321	315	0.988	173	432	14	1.371	2.497	10.61
LG3.2	350	341	0.988	223	536	16	1.572	2.404	14.19
LG5.1	346	342	0.989	235	528	12	1.544	2.247	18.13
LG1.1	361	354	0.99	222	476	10	1.345	2.144	11.62
LG5.2	371	367	0.992	202	412	9	1.123	2.04	8.36
LG2.2	371	369	0.993	235	398	7	1.079	1.694	12.29
LG4.2	376	370	0.993	210	374	5	1.011	1.781	7.98
LG4.1	379	373	0.992	224	448	7	1.201	2	6.97
LG3.1	393	384	0.989	261	622	14	1.62	2.383	14.26
LG2.1	392	390	0.991	245	544	9	1.395	2.220	12.03
LG1.2	418	411	0.991	264	596	14	1.45	2.258	10.34
LG3.1	497	485	0.99	353	952	14	1.963	2.697	17.83
LG4.2	571	553	0.99	410	1182	15	2.137	2.883	28.10
LG1.1	572	560	0.991	401	1046	12	1.868	2.608	19.13
LG5.1	591	570	0.986	417	1580	23	2.772	3.789	28.72
LG1.2	592	580	0.992	424	1034	11	1.783	2.439	20.32
LG2.2	597	580	0.989	423	1482	24	2.555	3.504	29.61
LG4.1	600	582	0.993	391	1000	14	1.718	2.558	18.08
LG5.2	636	615	0.991	425	1196	17	1.945	2.814	21.50
LG2.1	641	624	0.99	460	1434	25	2.298	3.117	24.28
LG3.2	655	643	0.992	458	1198	16	1.863	2.616	60.39
LG3.1	725	706	0.993	504	1378	14	1.952	2.734	21.86
LG1.2	732	708	0.989	542	2086	21	2.946	3.849	28.12
LG1.1	740	716	0.989	543	1986	19	2.774	3.657	27.05
LG4.2	777	755	0.99	574	2074	24	2.747	3.613	20.13
LG2.1	782	765	0.991	585	1952	22	2.552	3.337	23.98
LG5.1	782	768	0.992	578	1858	22	2.419	3.215	22.38
LG4.1	794	772	0.992	579	1808	16	2.342	3.123	24.83
LG5.2	787	773	0.989	566	2310	23	2.989	4.081	29.54
LG3.2	794	775	0.992	574	1918	20	2.475	3.341	24.15
LG2.2	794	777	0.991	608	2048	19	2.636	3.368	27.25

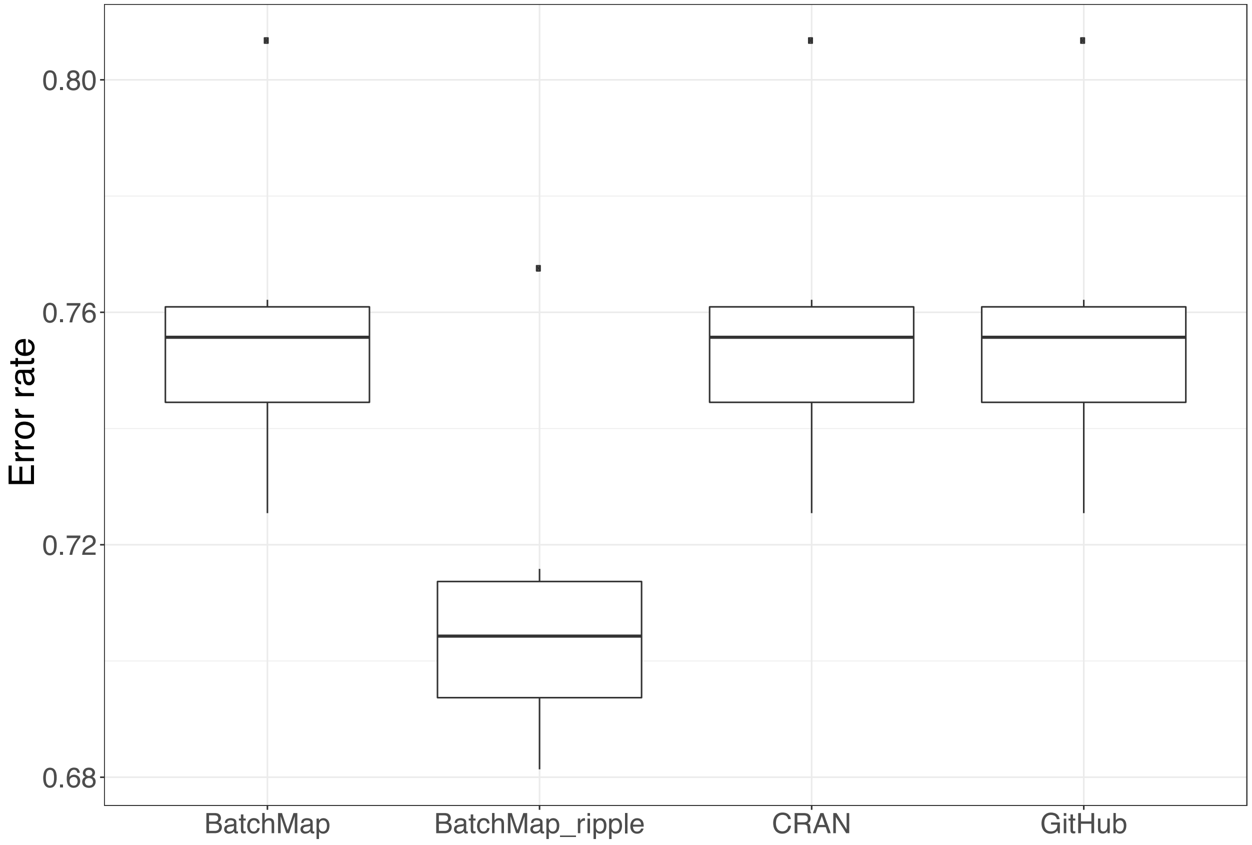

Figure C: Error rates (see equation 3) of all evaluation runs. OneMap (CRAN and GitHub versions) and BatchMap (regular and ripple versions) were run of each of the pseudo-testcrosses obtained from three linkage groups of the sim20k dataset (see Supplementary File Dataset_simulated_20k.txt). N = 6

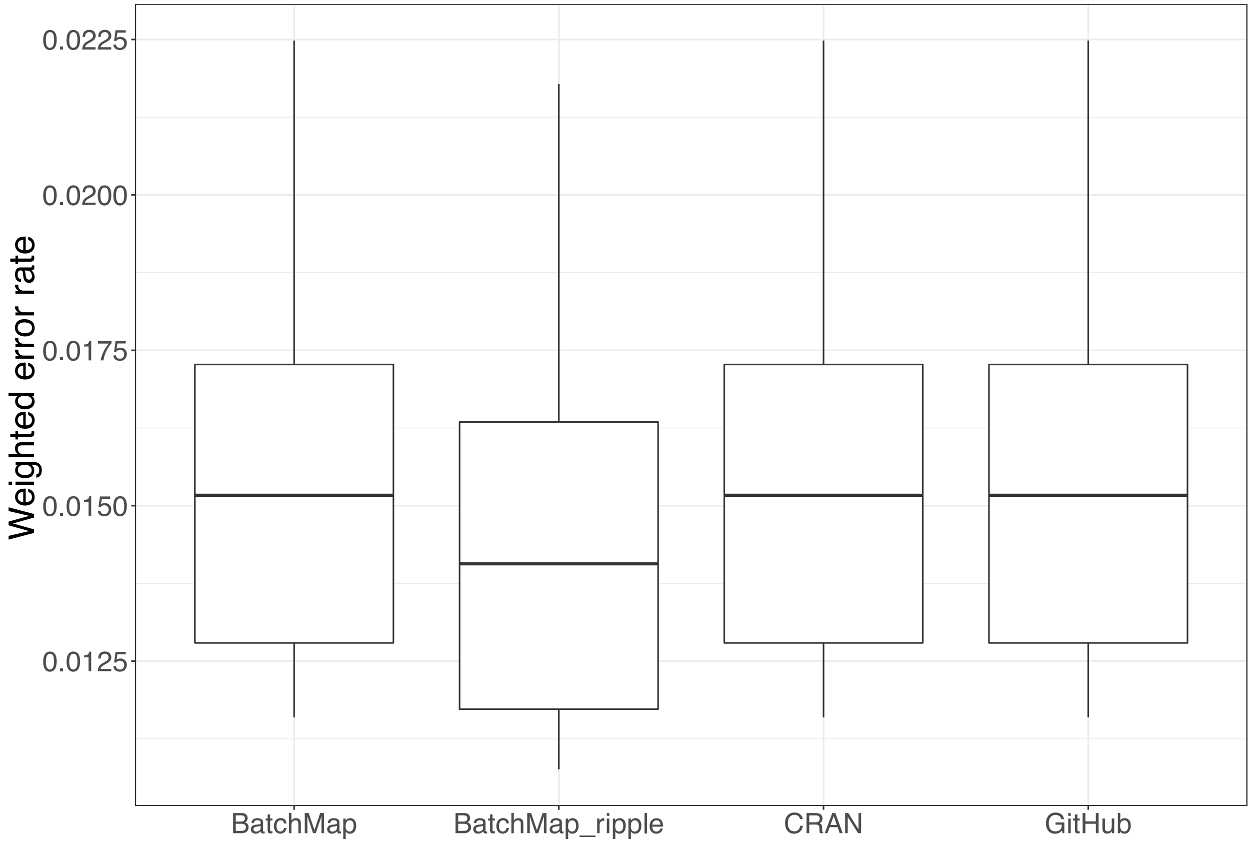

Figure D: Weighted error rates (see equation 4) of all evaluation runs. OneMap (CRAN and GitHub versions) and BatchMap (regular and ripple versions) were run of each of the pseudo-testcrosses obtained from three linkage groups of the sim20k dataset (see Supplementary File Dataset_simulated_20k.txt). N = 6

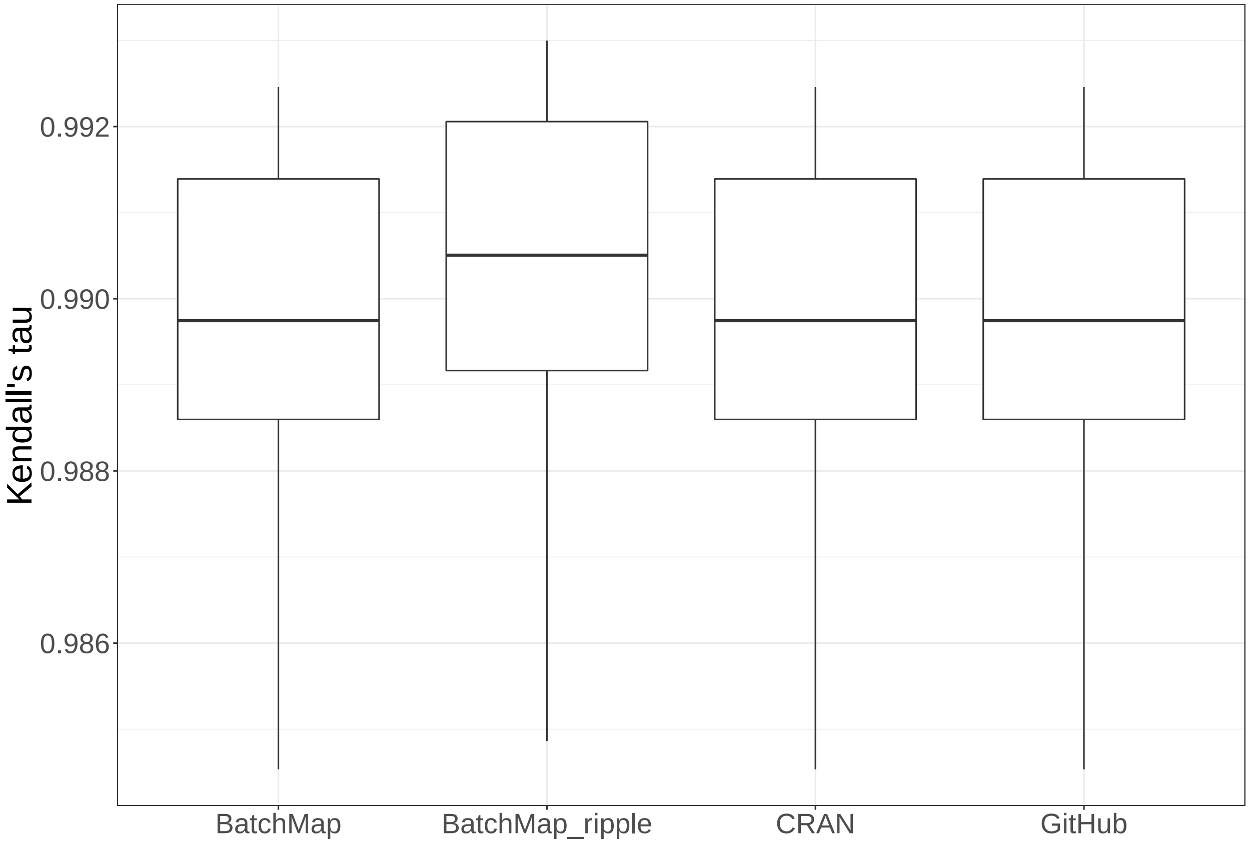

Figure E: Kendall's tau of all evaluation runs. OneMap (CRAN and GitHub versions) and BatchMap (regular and ripple versions) were run of each of the pseudo-testcrosses obtained from three linkage groups of the sim20k dataset (see Supplementary File Dataset_simulated_20k.txt). N = 6
